# Editorial Letter for Thematic Issue on Shoulder Pathology

**DOI:** 10.2174/1874325001711010064

**Published:** 2017-02-28

**Authors:** Guest Editor, Achilleas Boutsiadis

**Affiliations:** 424 General MIlitary Hospital Of Thessaloniki, Ring Road Egnatias, Efkarpia, 56429, Thessaloniki Greece; E-mail: boutsia@gmail.com

The high impact of common shoulder problems in the patient’s quality of life is already well established. Furthermore, a PubMed search with the keyword “shoulder” provides over 60,000 articles, reflecting the scientific interest on this area. The importance of this “Thematic Issue on Shoulder Pathology” is that it generated the idea of calling both residents, fellows and expert orthopaedic surgeons to a massive research, accumulation and sharing of high quality data.

For those who know the difficulty of preparing and writing a scientific article without any financial payments or other commercial benefits, we want to appreciate all the Guest Authors for their time and devotion that made this special issue possible. Additionally, we acknowledge all the reviewers for their hard work, through multiple revisions, in order to ensure the high quality of all articles. The Open Orthropaedics Journal and Bentham Science Publishers provided an outrageous, continuous and unrestricted support, for which we are appreciative.

We are now ready to present the outstanding results of this procedure and provide latest data on interesting topics like shoulder fractures, irreparable rotator cuff tears, application of biologics at the shoulder, suprascapular neuropathy, newer data in instability and shoulder rehabilitation.

We hope you like it.

